# Prevalence of potential risk of eating disorders among young, unprofessional European athletes: results of the ERASMUS+ project SCAED

**DOI:** 10.3389/fnut.2024.1398464

**Published:** 2024-10-15

**Authors:** Gordana Kenđel Jovanović, Tatjana Čulina

**Affiliations:** ^1^Department of Environment Protection and Health Ecology, Teaching Institute of Public Health of Primorsko-goranska County, Rijeka, Croatia; ^2^Department of Health Ecology, Faculty of Medicine, University in Rijeka, Rijeka, Croatia; ^3^Department of School and Adolescent Medicine, Teaching Institute of Public Health of Primorsko-goranska County, Rijeka, Croatia; ^4^Department of Family Medicine, Faculty of Medicine, University in Rijeka, Rijeka, Croatia

**Keywords:** adolescence, athletes, disordered eating, eating disorders, sport-related pressures

## Abstract

**Introduction:**

Adolescent athletes are at higher risk of developing eating disorders (ED) due to sports environment pressures and developmental characteristics. The ERASMUS+ project Sports Community against Eating Disorders (SCAED) aims to assess the prevalence of the potential risk of eating disorders among young, unprofessional European athletes and to provide them with easier access to professional support and knowledge.

**Methods:**

The online survey included 462 unprofessional athletes from six European countries aged 12–25 (average age 18.49 ± 5.50) on their socio-demographics, sports and lifestyle habits, behaviors, concerns, and perceived pressure from coaches and teammates regarding body weight and shape. The eating disorder potential risk was assessed using the Eating Disorder Examination for Adolescents (EDE-A) and Eating Disorders Screen for Athletes (EDSA).

**Results:**

Every seventh (14.9%, EDE-A) and fifth (19.9%, EDSA) of young, unprofessional European athletes were at possible risk for developing eating disorders. Overall potential risk (18.7% EDE-A, 26.6% EDSA), dietary restraint (12.9%), and concerns about eating (9.5%), shape (23.7%), and weight (19.6%) were significantly more prevalent among female athletes. Younger athletes showed a slightly higher prevalence and avoidance of food. Athletes training in weight-sensitive sports exhibited more behaviors related to eating disorders. Females (*p* = 0.003), younger, and those athletes training in less weight-sensitive sports noted higher weight- and shape-related pressure from coaches. Dissatisfaction with their current weight was expressed by 44.6% of athletes, while 46.1% thought that they needed to lose weight to improve performance.

**Conclusion:**

The observed prevalence of the potential risk of ED among young, unprofessional European athletes is concerning, particularly due to limited access to support. Efforts to reduce the prevalence should target female and younger athletes in weight-sensitive sports. The SCAED Erasmus+ project aims to decrease ED prevalence among young, unprofessional European athletes, their families, and coaches by supporting them through education and professional consultation.

## 1 Introduction

Eating disorders (ED) are psychiatric disorders classified by diagnostic criteria and are characterized by eating or weight-control abnormalities. They can lead to serious health problems if not recognized or treated (1). According to individual signs and degrees of severity, as detailed in the Diagnostic and Statistical Manual of Mental Disorders (Fifth Edition) (DSM-5) (2) and in the World Health Organization International Classification of Diseases, 11th Revision (ICD-11) (3), the EDs include anorexia nervosa, bulimia nervosa, binge eating disorder, and eating disorders not otherwise specified. Disordered eating (DE) is considered subclinical because regular diet-related behaviors, such as skipping meals, compulsive eating, compulsive exercise, and/or restrictive eating, are at a lower frequency or level of severity that fully meet the criteria for ED (4). Adolescence is a critical period of developmental vulnerability caused by increased focus on body image and appearance (5). It can be aggravated by the negative impacts of social media, on which adolescents spend a significant part of their time (6), especially on those social media that focus on image posting and viewing (7). Consequently, adolescence is a life period in which disordered eating and eating disorders are most frequently occurring (8), and also a period of developing self-worth based on eating, body weight, and shape, including restrictive or abnormal eating patterns (5).

Regular physical activity and organized sports participation among young adolescents have beneficial effects on their present and future health (9), such as higher bone mineral density in adulthood (10), decreased cardiovascular risk, overweight, obesity, and improvements in metabolic and cardiovascular parameters (11, 12), overall mental health (13), and subjective health later in young adulthood (14). Although sports participation positively influences overall physical and mental health (9), parental and coach pressure can jeopardize mental wellbeing, particularly by fostering the development of perfectionism (15, 16). Higher perfectionism is linked to eating disorder symptoms in children and adolescents (17). For example, athletes striving for perfection in performance may adopt unhealthy eating practices to meet perceived ideals (18). Young athletes may experience stress regarding balancing educational and social responsibilities that may lead to mental health indices, such as depressive symptoms, anxiety symptoms, emotional symptoms, hyperactivity symptoms, conduct problems, peer problems, and prosocial behavior. Consequent unhealthy coping activities may include disordered eating (19). Exposure to the pressure to conform to sporting body ideals can lead to unhealthy eating behaviors and body image issues (20, 21). Adolescents participating in organized sports may face additional stressors due to societal and sports-specific expectations if their psychophysiological changes during adolescence do not align (2, 9, 16, 22). A recent Lithuanian study revealed age- and sex-based differences; adolescent female athletes reported greater sociocultural pressure from family and peers and sports pressures from the coaches but also had a less positive image of their body compared with adult female athletes (23). Adolescent male athletes showed prevalent ED compensatory behaviors, such as vomiting, use of laxatives and diuretics, and excessive exercise, compared with adult athletes (23). Recently, it was estimated that the global proportion of children and adolescents with disordered eating is 22.36% (24). Among young elite athletes, the prevalence of EDs varies significantly due to differences in gender, location, types of sports, and self-reported screening tools. This wide heterogeneity greatly limits the ability to compare and interpret ED prevalence data (25). Still, it is highlighted that the most reliable prevalence of young athletes with an ED meeting DSM-4 criteria ranged from 5.6 to 7% (25). Furthermore, recent German research presented a prevalence of clinical eating pathology of 5.5% among elite adolescent athletes, with adolescent female athletes showing the highest rate of 9.6% (26). The existing data on the prevalence of eating disorders primarily focus on young elite athletes. Therefore, more research is necessary to understand the prevalence and behaviors associated with eating disorders among young unprofessional athletes who may not have the same level of support as elite athletes.

All the presented data emphasize adolescence as a critical period for early ED onset, the risk factors for ED development that adolescents are exposed to, and the observed rising prevalence. Therefore, efficient prevention activities, identification, and management approaches are needed in sporting environments. Based on their level of commitment, some unprofessional young athletes may be highly active, and compete nationally and internationally, similar to professional athletes (27). The difference is that professional athletes are often supported by a core multidisciplinary team constantly working on their performance and health (28), whereas unprofessional athletes rely more on support from their coaches and families. Although everyone working with athletes has an opportunity to identify early ED signs (4), coaches and families are in a unique position, which is important since early detection and shorter illness duration before admission to treatment are likely associated with better outcomes (29). European Education and Culture Executive Agency (EACEA)—Erasmus+, EU Solidarity Corps supported a project the Sports Community Against Eating Disorders (SCAED)(Project 101048829—SCAED—ERASMUS-SPORT-2021-SCP)^1^ with a basic aim to assess the prevalence and characteristics of ED among European unprofessional younger athletes and factors associated with the development and prevalence of ED in athletes. Partners from Bulgaria, Croatia, Greece, Italy, North Macedonia, and Poland designed the SCAED project intending to raise awareness about the current problems of ED among athletes, assess its size, and identify factors lying in the development background of ED. The findings of the project survey will help in developing reactive materials (Manual and Map of European Entities) for easier access to knowledge and professional support, specifically for coaches and athletes’ families. Since the analysis of factors associated with the development and prevalence of ED in athletes is important for preventing ED, the purpose of this study was to investigate the prevalence of potential risk for ED and risk factors associated with the development of ED among unprofessional European athletes of younger age. Although it is recognized that the prevalence of eating disorders is higher among female athletes (4), a scoping review from 2020 found that male athletes are also at risk for eating disorders and disordered eating behaviors, with up to 32.5% of adult male elite athletes experiencing eating disorders, with a higher prevalence in weight-sensitive sports (30). However, better understanding and standardized tools for the assessment and treatment of eating disorders in male athletes are needed, especially because of the noted rising prevalence. Based on the existing literature, it is assumed that the prevalence of ED, body image concerns, and sports-related pressures regarding weight and shape will be higher among female and younger athletes playing weight-sensitive sports.

## 2 Materials and methods

### 2.1 Procedure

This cross-sectional study used a convenience sampling method to explore the prevalence of possible risk of eating disorders and associated risk factors among unprofessional European athletes aged 12–25. National and local sports organizations from Bulgaria, Croatia, Greece, Italy, North Macedonia, and Poland were contacted and presented with the project’s aim. They were asked to contact coaches of all unprofessional sports clubs or school sports clubs that are members of national or local sports organizations to participate in the research. Coaches received the email with an invitation to participate in the study, a participant information form with project aim and details, and a link to the online survey. They were asked to email their athletes aged 12–25 years and their parents with an invitation to participate in the research. They received an online survey link separately for each athlete and one parent or guardian of the athlete. The inclusion criteria were unprofessional athletes aged 12–25 years and members of a school sports club or sports club. The exclusion criteria were professional athletes, younger than 12 years, older than 25 years, not members of a school sports club or sports club, and not fully completing the online questionnaire. The anonymous online survey was completed using Google Forms from September to December 2022. Before starting the survey, participants were informed about the research aims and content and informed that no identifying information would be collected. All the participants were asked to provide consent to participate by marking one option, “I agree to participate” or “I disagree to participate.” Those who disagreed were acknowledged, and the survey was completed; those who agreed were provided with an online survey. Participation was voluntary, and only the researchers had access to the data. All surveys had questions about sociodemographic variables, lifestyle, and risk behaviors related to eating disorders, and screening questionnaires for eating disorders. The Ethics Committee of the Teaching Institute of Public Health of Primorsko-goranska County approved this study.

### 2.2 Participants

For the purpose of this study, only the responses from the athletes will be presented, and only those from the surveys that the athletes completely answered entered into further analyses. Of the 3,258 surveys received from athletes, 462 (14.2% response rate) were completely answered. Of these, there were significantly more female athletes (52.2%) than male athletes (47.8%, *p* < 0.003), and more athletes younger than 18 years (59.7%, *p* = 0.127) (Table 1). The average age of participants was 18.49 ± 5.50 years, and their body mass index was 21.69 ± 3.31 kg/m^2^, ranging from 15.62 to 39.61 kg/m^2^. Significantly, most athletes had normal weight (75.1%, *p* < 0.001). There were significantly more athletes training in less weight-sensitive sports (*p* < 0.001), with mostly male athletes (*p* = 0.020), whereas female athletes were more prevalently trained in weight-sensitive sports (*p* = 0.020). Regarding age groups, younger athletes were more represented in both sports groups based on weight sensitivity (*p* = 0.385). Significantly highest proportion of athletes trained for 10 or more hours per week (*p* < 0.001). Daily breakfast consumption was reported by 53.0% of athletes, more male than female athletes (*p* = 0.067), and significantly more common among older athletes (*p* = 0.002) (Table 1). Regular intake of dietary supplements (vitamins, minerals, multivitamins/minerals, proteins, omega-3 fatty acids) was reported by 39.6% (*p* < 0.001) of athletes, more males than females (*p* = 0.218), and more by younger athletes (*p* = 0.271).

**Table 1:** Characteristics of 462 unprofessional European athletes aged 12–25 years.

Variables		*N*	%	*p*-value^*^
Sex	Males	221	47.8	0.003
	Females	241	52.2	
Age (years)	<18	276	59.7	0.127
	≥18	186	40.3	
Nutritional status	Underweight	59	12.8	<0.001
	Normal weight	347	75.1	
	Overweight and obesity	56	12.1	
Sports based on weight sensitivity	Weight-sensitive	163	35.3	<0.001
	Males	66	40.5	0.020
	Female	97	59.5	
	<18 years	93	57.1	0.385
	≥18 years	70	42.9	
	Less weight-sensitive	299	64.7	<0.001
	Males	155	51.8	0.020
	Female	144	48.2	
	<18 years	183	61.2	0.385
	≥18 years	116	38.8	
Training frequency (h/week)	10 or more	170	36.8	<0.001
	7–9	99	21.4	
	5–6	96	20.8	
	3–4	72	15.6	
	1–2	25	5.4	
Daily breakfast habits	All	245	53.0	0.193
	Males	127	57.5	0.067
	Females	118	49.0	
	<18 years	130	47.1	0.002
	≥18 years	115	61.8	
Regular dietary supplement intake	All	183	39.6	<0.001
	Males	94	42.5	0.218
	Females	89	36.9	
	<18 years	115	41.7	0.271
	≥18 years	68	36.6	

^*^Chi-square test (categorical variables).

### 2.3 Study methods

Based on self-reported body weight (kg) and height (cm), the body mass index (BMI) (kg/m^2^) was calculated. According to the criteria recommended by the WHO for those ≥18 years and older (31) and the International Obesity Task Force (IOTF) cut-offs for those <18 years (32), all athletes were classified into the underweight, normal weight, overweight, and obesity groups.

According to the proposed classification of sports based on weight sensitivity, all athletes were classified into weight-sensitive (WS) and less weight-sensitive (LWS) sports based on the sports in which they were training. The WS sports were sports judged esthetically, such as gymnastics, figure skating, competitive dancing, and synchronized swimming; endurance/gravitational sports, such as swimming, athletics, and cycling; weight class sports, such as boxing and martial sports. The LWS sports were sports such as ball games such as handball, basketball, football, and volleyball; high mass sports such as skiing, hockey, and discus; and technical sports such as fencing, archery, and shooting (33).

To assess the prevalence of ED among unprofessional athletes, two questionnaires were used. The first was the 36-item Eating Disorder Questionnaire for Adolescents (EDE-A), which assessed four risk behaviors (dietary restraint, eating concerns, shape concerns, and body weight concerns) in the past 14 days. It is an adapted version of the Eating Disorder Examination Questionnaire (EDE-Q) (34). Participants were asked to rate questions on a scale from 0 (no days/not at all/none of the times) to 6 (every day/markedly/every time) for the number of days in the previous 14. Questions answered with “No” rated with 0 and “Yes” with 1. The subscale scores were summed to obtain the global score. Each score has a maximum of six points, and higher subscale scores and global scores represent more problematic eating habits and attitudes. For evaluating individuals at potential risk of developing ED, a cutoff value greater than 2.8, which was clinically significant in most studies for detecting probable risk of ED (35), was used. Since the EDE-Q was initially developed within the female population, the possibility of ED in males may have been under-identified, so a cutoff value for male athletes was 1.68 (36). In this study, Cronbach’s *α* was 0.96, 0.94 in males, and 0.97 in females.

The EDE-A questionnaire is relatively extensive, containing 36 questions, and it is primarily intended to assess the general population. Therefore, the Eating Disorders Screen for Athletes (EDSA) as a short, validated screening tool for ED containing six questions and aimed at athletes of both sexes was also applied as a second questionnaire (37). The intention was to test a short questionnaire for its suitability and ease as a screening tool for the possible risk of ED among athletes. The EDSA consists of six questions; two relate to weight/shape concerns, and four relate to the importance of body weight, weight concerns, overeating concerns, and dietary restriction. Responses were recorded on a 5-point Likert-type scale with options of 1 (never), 2 (rarely), 3 (sometimes), 4 (often), and 5 (always). The total questionnaire results represent the average answers to all 6 items. The clinically significant cutoff score for the EDSA questionnaire was 3.33 (37) and was used in this study to indicate the possible risk of ED. The results of the EDE-A and EDSA questionnaires should be interpreted with caution because they represent screening tools for the possibility of ED and are not diagnostic criteria (36). Therefore, the global scores of both questionnaires were interpreted as measures of the potential individual risk of ED among young, unprofessional European athletes in this study. In this study, Cronbach’s *α* was 0.88, 0.84 in males, and 0.90 in females.

This study assessed potential risk factors for ED associated with weight-control behaviors. The athletes answered the following questions (yes/no): (1) whether they had three or more injuries in the last season or whether they had to finish the season early due to injury or illness; (2) were they worried about gaining weight during the off-season or when they are on sick leave; (3) are they satisfied with their current weight; (4) opinion about if they need to lose weight; (5) whether someone who is not a health professional told them to lose weight; (6) are they following a certain diet plan to achieve the best weight for their performance. For these six questions, Cronbach’s *α* was 0.66, 0.58 in males, and 0.71 in females. For this study, perceived pressure from a coach and teammates regarding diet and weight/body shape was assessed with eight following questions: (1) “I talk about food and diet with my teammates”; (2) “It makes me nervous that my coach controls my weight”; (3) “I compare myself to my teammates regarding my weight”; (4) “It bothers me when my coach asks me to weigh myself often”; (5) “It bothers me when my coach talks about my weight”; (6) “I feel uncomfortable when my coach and teammates talk about my weight and body shape”; (7) “I do not feel good when my teammates talk about my body”; (8) “I feel good if I weigh less than my teammates.” The athletes gave binary answers (yes or no). Score 1 was given for “no” to the first question, and “yes” to the remaining seven questions. The pressure score ranged from 0 to 8, where a score ≥ 5 indicated high pressure from the coach and teammates; coach pressure was based on questions 2, 4, 5, and 6, while teammates’ pressure was based on questions 3, 6, 7, and 8. This study assessed the dietary habits of athletes based on their recorded average frequency and quantity consumption of the listed foods and drinks during the previous week. They recorded the frequency intake as a choice between “never,” “once a week,” “2–3 times a week,” “4–5 times a week,” “6 times a week,” “once a day,” or “several times per day.” Each listed food or beverage had an associated median serving size, so the athletes recorded their average intake as a choice between “less than average,” “average,” or “more than average.” They also noted their breakfast eating habits. Because this study included young, unprofessional athletes from six European countries, due to sociocultural differences, this study assessed the athletes’ dietary adherence to the EAT-Lancet guidelines for a healthy and sustainable diet (38). The EAT-Lancet index consists of 14 food components in the EAT-Lancet reference diet of 2,500 kcal/d, including seven positive components (whole grains, vegetables, fruits, legumes, nuts, fish, and unsaturated oils) and seven negative components (potatoes, dairy, beef and lamb, pork, poultry, eggs, and added sugar). The score for each component ranged from 0 (no adherence) to 3 points (high adherence), representing a positive score for emphasized intake and a negative score for limited intake. The total possible EAT-Lancet index score can range from 0 (no adherence) to 42 points (perfect adherence, score 14 × 3 points). For this study, a total score of 14 was considered low adherence to the EAT-Lancet diet, a score of 15–27 was considered moderate adherence, and a score ≥ 28 was considered high adherence. First, the athletes’ absolute daily consumption of each index component in grams was divided by the individual total daily energy intake in kilocalories. Then, the grams per kcal-ratio were multiplied by 2,500. Each component was coded based on the cut-off values: 0 points when the cutoff value was not met or 1 point when the cutoff value was met (39).

### 2.4 Statistical analysis

Descriptive statistics were calculated, and the distribution of continuous variables was tested for normality in a preliminary analysis. The chi-square test was used to assess the associations between pairs of categorical variables. An independent-sample *t*-test was applied to compare the results of scores in the age, sex, and sport-weight sensitivity groups. Cronbach’s *α* was calculated to assess the internal consistency of the measurement questionnaire results. Binary logistic regression was applied to test the independent multivariable adjusted effects of sex, age, and sport-weight sensitivity groups on any occurrence of ED during the last 14 days, assessed separately for each of the two applied ED questionnaires. A *p*-value of less than 0.05 was considered statistically significant. All statistical analyses were performed using Statistica 12.7 for Windows (Statsoft Inc., Tulsa, OK, United States).

## 3 Results

### 3.1 Prevalence of potential risk factors of eating disorders according to age, sex, and weight sensitivity

Table 2 presents any occurrence frequencies of behaviors related to eating disorders during the last 14 days regarding age group, sex, and weight sensitivity sports group assessed using the Eating Disorder Questionnaire for Adolescents (EDE-A) and the Eating Disorders Screen for Athletes (EDSA). Through the application of questionnaires, athletes at potential risk of eating disorders can be detected. Based on the EDE-A questionnaire, the overall prevalence of those at potential risk was 14.9%, which was significantly higher in women than in men (*p* = 0.019) (Table 2). A slightly higher prevalence was observed among athletes younger than 18 years and those training in weight-sensitive sports, although without statistical significance. Regarding age groups, there was no significant difference in the reported frequencies of behaviors related to eating disorders assessed using the EDE-A questionnaire. Dietary restraint was almost twice as prevalent among women as men (*p* = 0.029). Significantly more women than men reported concern about eating (*p* < 0.001), shape (*p* < 0.001), and weight (*p* < 0.001). Female athletes also reported exercising significantly more than males (*p* = 0.033) to control shape and weight. After stratification into groups according to weight-sensitive sports, no significant difference was observed in the occurrence of risk factors for eating disorders (Table 2). Athletes training in weight-sensitive sports more commonly reported behaviors such as dietary restraint, binge eating, self-induced vomiting, and excessive exercise, whereas athletes training in less weight-sensitive sports more frequently reported concerns about weight and body shape. Both sports groups reported laxative and diuretic misuse at similar frequencies. All mentioned differences were not significant between the groups of sports based on weight sensitivity (Table 2).

**Table 2:** The prevalence of eating disorders and the occurrence (%) of any eating disorder risk behavior according to age, sex, and weight sensitivity sports groups during the last 14 days, assessed with the Eating Disorder Questionnaire for Adolescents (EDE-A) and the Eating Disorders Screen for Athletes (EDSA) in 462 European unprofessional athletes of younger age.

	All (*n* = 462)	<18 years (*n* = 276)	≥18 years (*n* = 186)	*p*-value^a^	Males (*n* = 221)	Females (*n* = 241)	*p*-value^b^	WS sport (*n* = 163)	LWS sport (*n* = 299)	*p*-value^c^
The Eating Disorder Questionnaire for Adolescents (EDE-A)										
Dietary restraint	9.9	10.1	9.7	0.869	6.8	12.9	0.029	12.3	8.7	0.216
Eating concern	5.6	6.5	4.3	0.310	1.4	9.5	<0.001	5.5	5.7	0.942
Shape concern	16.2	18.5	12.9	0.111	8.1	23.7	<0.001	14.7	17.1	0.516
Weight concern	13.2	14.9	10.8	0.201	6.8	19.1	<0.001	12.9	13.4	0.881
Binge eating	16.9	15.6	18.8	0.362	19.9	14.1	0.096	18.4	16.1	0.519
Self-induced vomiting	3.5	2.9	4.3	0.419	2.3	4.6	0.176	4.3	3.0	0.471
Laxative misuse	1.7	1.4	2.2	0.571	1.4	2.1	0.555	1.8	1.7	0.895
Diuretics misuse	1.3	0.0	3.2	0.003	0.0	2.5	0.018	1.2	1.3	0.920
Excessive exercise	27.7	27.2	28.5	0.758	23.1	32.0	0.033	32.5	25.1	0.088
At risk	14.9	16.7	12.4	0.203	10.9	18.7	0.019	15.3	14.7	0.856
The Eating Disorders Screen for Athletes (EDSA)										
Weight/shape/body composition self-confidence	23.6	23.9	23.1	0.844	20.8	26.1	0.178	27.0	21.4	0.133
Weight/shape/body composition dissatisfaction	19.9	18.5	14.5	0.265	14.0	19.5	0.116	18.4	16.1	0.519
Weight/shape/body composition concern	21.4	19.9	23.7	0.338	20.4	22.4	0.593	24.5	19.7	0.229
Leanness desire	14.7	15.9	12.9	0.366	7.7	21.2	<0.001	16.0	14.0	0.581
Concerns about eating	11.9	10.9	13.4	0.403	8.6	14.9	0.036	12.9	11.4	0.632
Food avoidance	23.4	27.5	17.2	0.010	22.6	24.1	0.715	20.2	25.1	0.240
At risk	19.9	21.7	17.2	0.231	12.7	26.6	<0.001	18.4	20.7	0.549

a Differences between age groups among all European unprofessional athletes of younger age.

b Differences between male and female European unprofessional athletes of younger age.

c Differences between weight-sensitivity sports groups among all European unprofessional athletes of younger age.

WS sport—weight-sensitive; LWS sport—less weight-sensitive sport.

The prevalence of athletes at potential risk of eating disorders assessed using the EDSA questionnaire was slightly higher (19.9%, Table 2). The prevalence was significantly twice as high among female athletes as male athletes (*p* < 0.001). There was no significant difference between the age and weight-sensitivity groups, but it was a little higher among younger athletes and among athletes training in less weight-sensitive sports. Regarding age groups, athletes younger than 18 years avoided food 1.6 times more than those older than 18 years (*p* = 0.010). The influence of weight, shape, or body composition on self-confidence was similar in both age groups. However, this caused dissatisfaction, which was slightly more among younger athletes (Table 2). Athletes younger than 18 years wanted to be leaner even though others thought differently more than older athletes who were more concerned about weight, shape, or body composition and had more frequent concerns over eating than younger athletes, but without significant differences. Similarly, the components of the EDSA questionnaire did not differ regarding weight-sensitivity sports groups, but compared with the LWS group, the WS group reported more frequently that their weight, shape, or body composition affected self-confidence, causing more dissatisfaction and concerns (Table 2). The WS group also wanted to be leaner even if others may have thought that they were already lean and reported more frequent concern over eating than the LWS group, whereas the LWS group more frequently reported avoiding foods that influenced their weight, shape, or body composition (Table 2).

### 3.2 Risk factors for ED associated with weight-control behaviors

**Figure 1:**
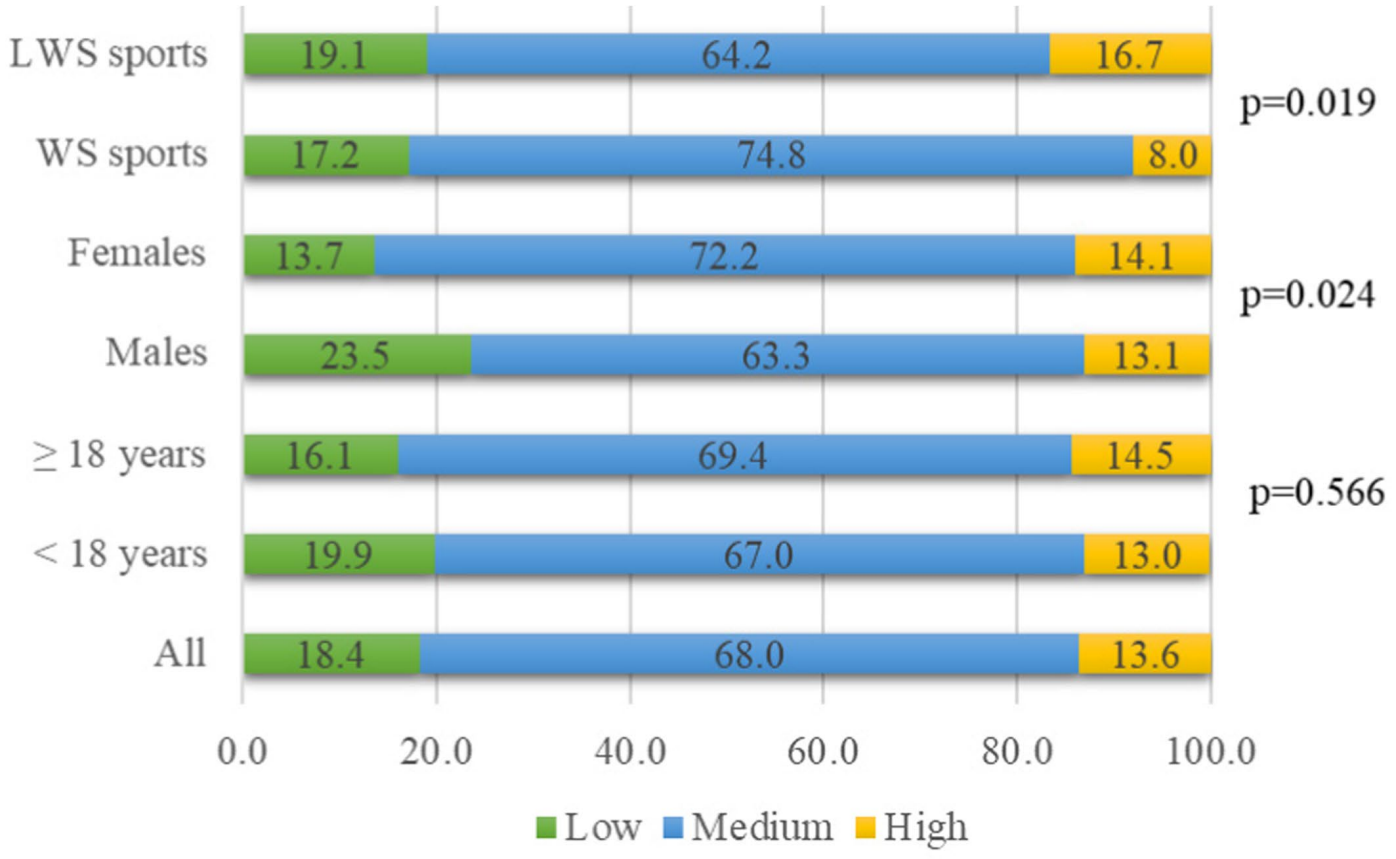
Adherence to EAT-Lancet diet in 462 European unprofessional athletes of younger age (%).

Table 3 presents the occurrence of risk factors for eating disorders associated with weight-control behaviors among unprofessional European athletes of younger age. Fifty-eight (12.6%) athletes reported having three or more injuries or finished training season earlier due to injury or illness, significantly more those who were older than 18 years (*p* = 0.037), and the results did not differ according to sex and weight-sensitivity sports groups. Nearly half (44.6%) of all athletes declared dissatisfaction with their current weight, which was significantly more common among older athletes (*p* < 0.001), and those who trained in less weight-sensitive sports (*p* = 0.001). Dissatisfaction with current weight did not differ according to sex (Table 3). Almost half of the participants (46.1%) thought that they needed to lose weight for better performance, significantly more by older athletes (*p* = 0.020), and those training less weight-sensitive sports (*p* = 0.003). A third of all athletes (27.7%) were told that they should lose weight by someone who is not a health professional, such as parents, friends, or teammates. Female athletes reported this significantly more (*p* = 0.033) than male athletes, and no significant differences were found according to age and weight-sensitivity sports groups. A third of athletes (35.3%) also reported worrying about gaining weight during the off-season or due to injury or sick leave; significantly more female athletes (*p* = 0.006), and there was no difference according to age and weight-sensitivity sports groups. Any negative experiences with weight loss were reported by 21.0% of the athletes, significantly more female athletes (*p* = 0.009). Regarding age groups, there was an almost an equal proportion of those with negative experiences, and a similar trend was noted for weight-sensitivity sports groups, but without statistical significance (Table 3). A third of athletes (33.5%) stated that they currently followed a specific dietary plan to achieve their desired body weight, more significantly older athletes (*p* < 0.001), and did not report differences according to sex and weight-sensitivity sports groups. Figure 1 presents the results of the athletes’ diet quality, which was evaluated as adherence to the EAT-Lancet diet (39). Most athletes had a diet that moderately adhered to the EAT-Lancet diet, with 14% of those whose diet was highly adhered to (*p* < 0.001). Significantly more female athletes (*p* = 0.024) had a diet that moderately and highly adhered to the EAT-Lancet diet. Athletes with a diet that highly adhered to the EAT-Lancet diet trained more in LWS sports; however, there was a higher proportion of athletes training in WS sports with a diet that moderately and highly adhered to the EAT-Lancet diet (*p* = 0.019).

**Table 3:** Occurrence (*n*, %) of risk factors for eating disorders associated with weight-control behaviors among 462 European unprofessional athletes of younger age regarding age, gender, and weight-sensitivity sports groups.

Risk factors	All (*n* = 462)	<18 years (*n* = 276)	≥18 years (*n* = 186)	*p*-value^a^	Males (*n* = 221)	Females (*n* = 241)	*p*-value^b^	WS sport (*n* = 163)	LWS sport (*n* = 299)	*p*-value^c^
Three or more injuries or finished training season earlier due to injury or illness.	58 (12.6)	26 (9.4)	32 (17.2)	0.037	34 (15.4)	24 (10.0)	0.079	17 (10.4)	41 (13.7)	0.309
Not satisfied with current body weight.	206 (44.6)	58 (21.0)	148 (79.6)	<0.001	97 (43.9)	109 (45.2)	0.773	56 (34.4)	150 (50.2)	0.001
Think to need to lose weight for better performance.	213 (46.1)	115 (41.7)	98 (52.7)	0.020	106 (48.0)	107 (44.3)	0.443	60 (36.8)	153 (51.2)	0.003
Not a health professional told to lose weight.	128 (27.7)	71 (25.7)	57 (30.6)	0.246	51 (23.3)	77 (32.0)	0.033	52 (31.9)	76 (25.4)	0.137
Worries regarding weight gain during the off-season, injury, or sick leave.	163 (35.3)	93 (33.7)	70 (29.0)	0.385	64 (29.0)	99 (41.0)	0.006	62 (38.0)	101 (33.8)	0.360
Currently follow a specific diet plan to achieve the desired weight.	155 (33.5)	41 (26.4)	114 (61.3)	<0.001	67 (30.3)	88 (36.5)	0.159	62 (38.0)	93 (31.1)	0.132
Any negative experience with losing weight.	97 (21.0)	53 (19.2)	44 (23.7)	0.249	35 (15.8)	62 (25.7)	0.009	35 (21.5)	62 (20.7)	0.853

a Differences between age groups among all European unprofessional athletes of younger age.

b Differences between male and female European unprofessional athletes of younger age.

c Differences between weight-sensitivity sports groups among all European unprofessional athletes of younger age.

WS sport—weight-sensitive; LWS sport—less weight-sensitive sport.

### 3.3 Prevalence of sporting pressure from teammates and coaches

Table 4 presents the prevalence of perceived sporting pressure by athletes, including the pressure from coaches and teammates according to age, sex, and weight-sensitivity sports groups. Every ninth athlete (11.0%) declared experiencing sporting pressure from the coach and teammates, significantly 3 times more female athletes (*p* < 0.001), but no difference was observed between age groups and between weight-sensitivity sports groups. Nevertheless, higher sporting pressure perceived younger athletes (12.3%) and those training the LWS sports (11.7%), the difference was not significant. Half of the athletes perceived pressure from one or more situations regarding weight, shape, or diet, but the perception was not significantly different between the age, sex, or weight-sensitivity sports groups. Pressure from teammates was slightly more reported by younger athletes, female athletes, and those training less weight-sensitive sports, without significant difference. Situations perceived as pressure from the coach were reported by one-third of the athletes, statistically more female athletes (*p* = 0.003). There was no difference between age groups in the frequency of perceived pressure from the coach or weight sensitivity, although slightly more athletes training in LWS sports reported this pressure.

**Table 4:** Frequency (%) of perceived sporting pressure, pressure from teammates, and coaches regarding diet, weight, and body shape among 462 European unprofessional athletes of younger age.

	All (*n* = 462)	<18 years (*n* = 276)	≥18 years (*n* = 186)	*p*-value^a^	Males (*n* = 221)	Females (*n* = 241)	*p*-value^b^	WS sport (*n* = 163)	LWS sport (*n* = 299)	*p*-value^c^
Sporting pressure	11.0	12.3	9.1	0.285	5.4	16.2	<0.001	9.8	11.7	0.536
Teammates pressure	51.5	54.0	47.8	0.196	47.1	55.6	0.066	46.0	54.5	0.069
Coach pressure	30.7	30.4	31.2	0.864	24.0	36.9	0.003	26.4	33.1	0.134

a Differences between age groups among all European unprofessional athletes of younger age.

b Differences between male and female European unprofessional athletes of younger age.

c Differences between weight-sensitivity sports groups among all European unprofessional athletes of younger age.

WS sport—weight-sensitive; LWS sport—less weight-sensitive sport.

### 3.4 Effects of sex, age, and weight sensitivity on the odds of the occurrence of eating disorders

Assessed with the EDE-A questionnaire and compared with male athletes, female athletes were significantly two times more likely to report dietary restraint in the last 14 days (*p* = 0.016) (Table 5), almost eight times more likely to be concerned about eating (*p* < 0.001), and three times more likely to report shape (*p* < 0.001) and weight concerns (*p* < 0.001), and one and half times more likely to report excessive exercise to reduce body weight (*p* = 0.017). Male athletes were two times more likely to report binge eating than female athletes (*p* = 0.002) (Table 5). Regarding age groups, there was no significant difference in the odds of reporting any behaviors related to eating disorders, and similarly, there was no difference in weight-sensitive sports, except for excessive exercise, which athletes training weight-sensitive sports reported more often (*p* = 0.044). The odds for reporting behaviors assessed with the EDSA questionnaire (Table 6) were significant regarding sex, where female athletes were significantly two times more likely to report weight, shape, or body composition concern (*p* = 0.011) and concern over eating (*p* = 0.019). Additionally, they were three times more likely to report a desire to be leaner in addition to that others think differently (*p* < 0.001) than male athletes. Athletes older than 18 years were almost twice as likely (*p* = 0.014) to report weight, shape, or body composition concerns, whereas 55% were less likely to report food avoidance (*p* = 0.005). There were no significant differences in the odds of reporting behaviors for ED assessed using the EDSA questionnaire regarding weight-sensitivity sports (Table 6).

**Table 5:** Effects of sex, age, and weight sensitivity on the odds ratios of eating disorders during the last 14 days assessed using the Eating Disorder Questionnaire for Adolescents (EDE-A) among 462 European unprofessional athletes of younger age.

		OR	95% CI	*p*-value
Dietary restraint	Male	1		
	Female	2.03	1.06–3.87	0.016
	<18 years	1		
	≥18 years	1.05	0.57–1.97	0.435
	Weight-sensitive sport	1		
	Less weight-sensitive sport	0.68	0.37–1.26	0.111
Eating concern	Male	1		
	Female	7.67	2.27–25.91	<0.001
	<18 years	1		
	≥18 years	0.64	0.27–1.51	0.157
	Weight-sensitive sport	1		
	Less weight-sensitive sport	1.03	0.45–2.37	0.471
Shape concern	Male	1		
	Female	3.49	1.98–6.15	<0.001
	<18 years	1		
	≥18 years	0.65	0.39–1.11	0.056
	Weight-sensitive sport	1		
	Less weight-sensitive sport	1.19	0.70–2.02	0.258
Weight concern	Male	1		
	Female	3.19	1.73–5.91	<0.001
	<18 years	1		
	≥18 years	0.69	0.39–1.22	0.102
	Weight-sensitive sport	1		
	Less weight-sensitive sport	1.04	0.59–1.84	0.440
Binge eating	Male	1		
	Female	0.44	0.26–0.76	0.002
	<18 years	1		
	≥18 years	1.26	0.77–2.05	0.181
	Weight-sensitive sport	1		
	Less weight-sensitive sport	0.85	0.51–1.40	0.260
Self-induced vomiting	Male	1		
	Female	2.07	0.71–6.04	0.092
	<18 years	1		
	≥18 years	1.51	0.56–4.09	0.211
	Weight-sensitive sport	1		
	Less weight-sensitive sport	0.69	0.25–1.89	0.236
Laxative misuse	Male	1		
	Female	1.54	0.36–6.52	0.279
	<18 years	1		
	≥18 years	1.49	0.37–6.05	0.287
	Weight-sensitive sport	1		
	Less weight-sensitive sport	0.91	0.21–3.85	0.447
Diuretics misuse	Male	1		
	Female	–		
	<18 years	1		
	≥18 years	–		
	Weight-sensitive sport	1		
	Less weight-sensitive sport	1.09	0.20–6.02	0.460
Excessive exercise	Male	1		
	Female	1.57	1.04–2.37	0.017
	<18 years	1		
	≥18 years	1.07	0.71–1.62	0.378
	Weight-sensitive sport	1		
	Less weight-sensitive sport	0.69	0.46–1.06	0.044

**Table 6:** Effects of sex, age, and weight sensitivity sport class on the odds ratios of eating disorders during the last 14 days assessed using the Eating Disorders Screen for Athletes (EDSA) among 462 European unprofessional athletes of younger age.

		OR	95% CI	*p*-value
Weight/shape/body composition self-confidence	Male	1		
	Female	1.13	0.73–1.77	0.292
	<18 years	1		
	≥18 years	0.96	0.62–1.48	0.422
	Weight-sensitive	1		
	Less weight-sensitive	0.71	0.46–1.11	0.067
Weight/shape/body composition dissatisfaction	Male	1		
	Female	1.48	0.91–2.44	0.059
	<18 years	1		
	≥18 years	0.75	0.45–1.25	0.133
	Weight-sensitive	1		
	Less weight-sensitive	0.85	0.51–1.40	0.260
Weight/shape/body composition concern	Male	1		
	Female	1.80	1.09–2.98	0.011
	<18 years	1		
	≥18 years	1.68	1.06–2.68	0.014
	Weight-sensitive	1		
	Less weight-sensitive	0.76	0.48–1.19	0.115
Leanness desire	Male	1		
	Female	3.22	1.80–5.77	<0.001
	<18 years	1		
	≥18 years	0.78	0.46–1.34	0.183
	Weight-sensitive	1		
	Less weight-sensitive	0.86	0.51–1.47	0.291
Concern about eating	Male	1		
	Female	1.87	1.04–3.36	0.019
	<18 years	1		
	≥18 years	1.27	0.72–2.24	0.202
	Weight-sensitive	1		
	Less weight-sensitive	0.87	0.49–1.55	0.316
Food avoidance	Male	1		
	Female	1.08	0.70–1.67	0.357
	<18 years	1		
	≥18 years	0.55	0.34–0.87	0.005
	Weight-sensitive	1		
	Less weight-sensitive	1.32	0.83–2.10	0.121

## 4 Discussion

### 4.1 Prevalence of the potential risk for eating disorders

The present study revealed that every seventh young, unprofessional European athlete aged 12–25 years reported behaviors of a possible eating disorder as assessed using the EDE-A and every fifth as assessed using the EDSA questionnaire, based on an online survey conducted in six European countries. These findings are consistent with data from similar studies that showed that approximately every fifth individual in late adolescence and early adulthood worldwide reports an eating disorder based on screening questionnaire scores above those recognized as “risk” cut-off scores (40). The present study’s prevalence of EDs is also in line with the prevalence of a meta-analysis of studies from 25 countries, which revealed that every eight high school students showed multiple indicators of problematic eating attitudes and behavior (41), and also in line with the reported prevalence and correlations of mental health symptoms or disorders in youth elite athletes aged 12–18 years (42). Both screening questionnaires that were applied in this study found a twice-higher significant prevalence of ED behavior in female athletes than in males, which is consistent with an acknowledged higher prevalence of ED among girls (41, 43), and a higher proportion of disordered eating among female children and adolescents at both the athletic and global levels (24, 40, 41).

### 4.2 Effects of sex, age, and weight sensitivity in sports on the prevalence of eating disorders

While earlier research focused more on females, recent studies indicate that EDs are no longer primarily a common female problem (43). Instead, they present differently in males, particularly as muscularity-oriented disordered eating (44). Moreover, disordered eating may frequently go unnoticed among boys (45), mainly due to the limitations of current diagnostic criteria for eating disorders (2). Boys tend to exhibit specific disordered eating behaviors, such as efforts to increase muscle mass and weight to counteract body image dissatisfaction (45), and also because boys are presumed to underreport having a problem because of the stigma related to female sex and DE (46, 47). In the present study, female athletes reported more eating, shape, and weight concerns, whereas binge eating was twice as frequently reported among male athletes. Female athletes were more frequently worried about losing control over their eating and its impact on their weight, shape, or body composition. This heightened concern among females could be explained by their greater anxiety about eating, weight, and shape and, a stronger desire to be thin, despite others’ perception. Gender disparities among athletes in body image dissatisfaction, weight, and shape concerns are well known, with a higher prevalence among female athletes (48, 49). Moreover, female athletes in this study more often expressed worries about gaining weight during the off-season or due to injury or sick leave compared to their male counterparts. Similar fear was more pronounced among athletes during the COVID-19 pandemic lockdown, where athletes emphasized that changes during that period to their exercise and training were the most significant factor affecting both their perception of their body and their relationship with food (50). Their actions resulted in increased binge eating, restriction, guilt, and shame, or increased/decreased food control (50). The latter concern was found to be more common to athletes in weight-sensitive sports (51), such as esthetic (e.g., gymnastics), weight class (e.g., boxing), gravitational endurance (e.g., long-distance running), and gravitational technical (e.g., high jump) sports, as the weight has been shown to have a significant influence on performance (52). Similar ED behaviors were also observed in the present study. Athletes training in weight-sensitive sports reported more often self-induced vomiting and excessive exercise, dietary restraint, and binge eating. They were also more often concerned about eating and their weight, shape, or body composition, as compared to athletes training in less weight-sensitive sports. Athletes who trained in less weight-sensitive sports were significantly more dissatisfied with their weight, particularly older athletes who more often followed a diet to achieve the desired weight for better athletic performance. Mixed-weight sport-specific differences in ED behaviors and body dissatisfaction were also previously observed and concluded that eating behavior disturbances were not limited to sports that emphasize leanness (53). When the competitive level was included, they were present, which was also shown by the recent systematic review and meta-analysis where the authors concluded that body image dissatisfaction is a general characteristic of females and athletes in esthetic sports (48). Furthermore, participating in competitive sports, rather than leisure exercise, in weight-sensitive sports may be somewhat protective against body image concerns during adolescence (53). Weight-sensitive sports often emphasize a lean body for performance or aesthetic reasons, which increases the pressure to maintain a low body weight or ideal body type, while sports involving weight classes may lead athletes to extreme weight controls to qualify for a particular category (54). Even in sports that are less focused on weight, such as team sports, high-performance demands or the competitive environment can still lead athletes to feel pressured to adhere to certain body standards or engage in unhealthy eating behaviors to fit in or to cope with stress (54). The lack of significant difference in the ED prevalence in the present study regarding weight sensitivity is also in line with other studies in addition to the different ED assessment tools used (23, 48, 53). There may be different reasons for eating disorders depending on the age group. Adolescence is a time of significant hormonal changes that can impact mood and behavior and potentially lead to the development of eating disorders, particularly in those genetically predisposed (55). Peer influence and social media can also play a role, as they often promote ideal body types, leading to body dissatisfaction and unhealthy eating behaviors (7). Among adult athletes, societal pressures to conform to beauty standards and maintain a youthful appearance can also lead to unhealthy behaviors (56). The present study found a similar but slightly higher prevalence among older athletes. Regarding age groups, behaviors related to eating disorders did not differ significantly in prevalence, although there were observed differences in their manner. The lack of a significant age difference can be explained by the possibility that a longer engagement in sports can influence a more positive view of sports health effects and, therefore, affect a more positive self-image and healthier dietary behavior.

### 4.3 Risk factors for eating disorders

It has been shown that athletes at more professional and organized sporting levels have higher body satisfaction (57, 58). Because body weight is not important for sports performance in less weight-sensitive sports, athletes in the included sports could have a higher body weight that they are not satisfied with, which can explain these results. Although the athletes in this online survey self-reported their body weight and height, no further calculations were made, as there could be possible bias in their under- or over-reporting, which could lead to wrong conclusions. Still, weight-sensitive sports athletes had significantly lower body weight than athletes of less weight-sensitive sports (data not shown). More female athletes participated in the weight-sensitive sports group, which could explain noticed higher body weight dissatisfaction in this group. A recent systematic review on the potential association of body image perception and/or body image satisfaction with dietary habits in adolescents (59), despite results heterogeneity, concluded that adolescents who underestimate their body weight tend to report unhealthier dietary habits. Conversely, those who overestimate or accurately perceive their body weight tend to follow healthier diets. Additionally, those driven for thinness may engage in either healthy or unhealthy weight loss behaviors. Gender-related divergences in body image and dietary habits reflect a desire among girls for a thinner body and among boys for a muscular one (59). In the present study, female athletes and those training in weight-sensitive sports reported healthier dietary habits, which agrees with a previously discussed systematic review (59). A study among German elite athletes aged 13–18 years showed that their mental associations of weight loss and success, along with perceived social pressure regarding eating and body shape, were strongly associated with their eating behavior and predicted symptoms of eating disorders (26). Nearly a third of the athletes in the present study, particularly female athletes, perceived social pressure to lose weight from non-professionals. Unsafe weight management practices, such as self-help or advice from individuals lacking expertise in sports nutrition and safe weight management, can lead to disordered eating, compromise athletic performance, and negatively impact an athlete’s health (60). About one-fifth of the athletes in the present study, with a higher prevalence among females, experienced negative health effects from weight loss. This underscores the need for providing evidence-based, personalized nutritional advice for young, unprofessional athletes from registered dietitian nutritionists. Recognizing the importance and skills of registered dietitian nutritionists experienced in sports nutrition is crucial for maintaining health, improving athletic performance, and preventing the development of ED among athletes. Consequently, many sports organizations have developed models to integrate them to work with athletes to improve their health (61, 62). This importance is even greater for unprofessional athletes whose level of training and competition is similar to that of the athletes of the national team, who mostly have the support of the core multidisciplinary team, including registered dietitians or nutritionists, and sports psychologists, especially when this study revealed that the prevalence of EDs is similar between these two groups of athletes, as shown with this study. They will likely be exposed to similar pressure from coaches, peers, and teammates.

There is a well-documented association between the pressure of coaches on the body image of athletes with their body image concerns and disordered eating (63). Similarly, pressure from teammates and peers regarding athletes’ body weight and appearance also plays a significant role (64). Coaches have a significant influence on athletes’ lives, and their comments about weight and appearance, together with those from teammates, can encourage disordered eating behaviors and disturbed attitudes toward nutrition (65). However, in this study, female athletes experienced the highest pressure related to weight and shape from their teammates, but significantly more from their coaches, which is consistent with the findings of previous studies (66–68). At the same time, there was no difference in perceived pressure according to age or weight-sensitive sport. These athletes were also more concerned and dissatisfied with their weight and shape, making them more likely to be screened and included in preventive programs since prevention and early diagnosis of eating disorders and disordered eating are crucial. Preventive education programs should focus on recognizing the symptoms of eating disorders, promoting a positive body image, healthy relationships with food, enhancing nutrition knowledge, and understanding the impact of nutrition on health and sports performance, which are especially important during adolescence (5, 9). It is essential to avoid negative comments and pressure on body weight and shape in all environments, including sports, peers, and families, and to provide access to experts who can advise athletes on nutrition and eating disorders (4, 68).

Although this study did not evaluate the socioeconomic status of athletes, it was shown that athletes’ socioeconomic status (SES) significantly influences their risk of developing eating disorders. Athletes with higher SES may have better access to nutritional education and resources, which helps them maintain healthy eating habits. They can also afford professional support, such as dietitians or specialized coaches, for a difference from those with lower SES athletes (69). Athletes from different SES backgrounds may face varying social and cultural pressures regarding body image, consequently potentially increasing the risk of eating disorders (54, 70). Based on the above-mentioned findings and this study’s results, the goals and actions of the Erasmus project SCAED are justified. The project aims to raise awareness of eating disorders within the sports community and provide young athletes, their families, and coaches with a manual and an interactive map of entities that can educate, help, and offer adequate support.

### 4.4 Strengths and limitations

The present study has certain strengths and limitations. The study found a significant prevalence of the potential risk of ED among young European athletes, which represents a worrying situation and highlights the need for future screening and preventive, educational, and supportive programs specifically for unprofessional athletes, their families, and coaches, such as the SCAED project aims and tasks. The study’s strengths also lay in its multicentric design and screening of a large sample of unprofessional athletes from six European countries and the inclusion of athletes of different types of sports. The study used two screening questionnaires, which both revealed a significant proportion of young, unprofessional European athletes at potential risk of ED, although it can be assumed that not all those at potential risk will develop clinically diagnosed ED. A short EDSA questionnaire has shown to be a suitable brief screening tool for the possible risk of ED. Both screening tools have certain limitations. The EDE-A may not effectively capture disordered eating in athletes due to the underreporting of symptoms, focus on weight and shape concerns, and lack of validation in athletic populations (71, 72). New screening tools such as the Eating Disorders Screen for Athletes (EDSA) and the Disordered Eating Screen for Athletes (DESA-6) have been developed and validated, showing higher sensitivity and specificity for athletes (37, 73). The EDSA is a tool designed to identify eating disorders in athletes, but it has several limitations. Primarily, it was validated using cross-sectional data, which means it does not account for changes over time. This limitation affects its ability to track the progression or remission of eating disorders. The EDSA may not fully capture the different ways eating disorders manifest in male and female athletes and may not adequately account for the unique pressures and behaviors associated with various sports. It was primarily validated in the USA, which may limit its applicability in different cultural contexts. Since there is a need for ongoing validation and adaptation of screening tools like the EDSA, this study’s results may add to the present knowledge. However, the cross-sectional design of the study restricts the possibility of establishing associations between the study variables and the potential risk of ED. The potential risk of ED and ED behaviors was evaluated using a self-assessment method. Thus, the participants’ subjective interpretations of the questions and reporting may have influenced the results, which should be interpreted with caution. Self-reporting of ED behaviors related to overeating is recognized as a problematic methodology because participants tend to be biased when assessing food quantity (74). Additionally, it is associated with shame and feelings of guilt, which can affect the participants’ responses (35). Validated and specialized tools to screen for disordered eating and ED in athletes are limited in number and quality; therefore, self-reported tools should be followed by an interview-based assessment using sport-specific or general approaches, if possible (4). This survey had a low response rate, possibly because of athletes’ low interest in the study subject, the survey length, and its topic, and not those athletes who did not have an email to receive a link with a questionnaire. It is acknowledged that this may lead to sampling bias and limit the generalizability of the study findings. However, this study used fully completed questionnaires to compare the results and determine the validity and reliability of both screening tools that were used for assessing the possible risk of the prevalence of eating disorders. The length of the online form may have caused greater withdrawal and incomplete completion; therefore, the inclusion of more participants in future studies with similar aims may affect the observed results of this survey.

## 5 Conclusion

A survey conducted among young, unprofessional European athletes found that every seventh and fifth of them were at possible risk for developing eating disorders, depending on the screening questionnaire for ED that was used. This finding is consistent with similar studies conducted among athletes and the general population. The highest risk groups were female athletes and those under 18 years of age. Their primary concern is their body weight, shape, and/or composition, and the impact it has on their self-confidence. Female athletes perceived body dissatisfaction and sporting pressure related to weight and shape more than others. A third of athletes currently follow a weight-loss diet, and older athletes twice as much as others, which may be risk factors for disordered eating (26). The survey results present a worrying situation among young, unprofessional European athletes, especially because they have limited access to multidisciplinary teams and the support that professional athletes have. Therefore, future efforts to diminish the observed and rising prevalence of eating disorders among athletes should specifically be aimed at unprofessional athletes, particularly female and younger athletes, and those training in less weight-sensitive sports. Those actions should include education, professional consultation (psychologist, nutritionist), and support involving coaches and families. Since the SCAED Erasmus+ project goal is to reduce the prevalence of ED and related risk behaviors by providing education and support to young and unprofessional European athletes, their families, and coaches, taking those actions can decrease the prevalence of ED among young European athletes.

## Data Availability

The original contributions presented in the study are included in the article/supplementary material, further inquiries can be directed to the corresponding author.

## Footnotes

^1^https://scaed.eu/
